# Supplementary material for: Src inhibition attenuates polyglutamine-mediated neuromuscular degeneration in spinal and bulbar muscular atrophy
Source: Nat Commun. 2019 Sep 19;10:4262. doi: 10.1038/s41467-019-12282-7 (PMC6753158; doi:10.1038/s41467-019-12282-7)
Supplement: Supplementary file 1 — Supplementary Information [file 41467_2019_12282_MOESM1_ESM.pdf]

Src inhibition attenuates polyglutamine-mediated neuromuscular degeneration in spinal and bulbar muscular atrophy.

Madoka Iida, Kentaro Sahashi, Naohide Kondo, Hideaki Nakatsuji, Genki Tohnai, Yutaka Tsutsumi, Seiya Noda, Ayuka Murakami, Kazunari Onodera, Yohei Okada, Masahiro Nakatochi, Yuka Tsukagoshi Okabe, Shinobu Shimizu, Masaaki Mizuno, Hiroaki Adachi, Hideyuki Okano, Gen Sobue, Masahisa Katsuno

Supplemental Figures 1-19

Supplementary Table 1

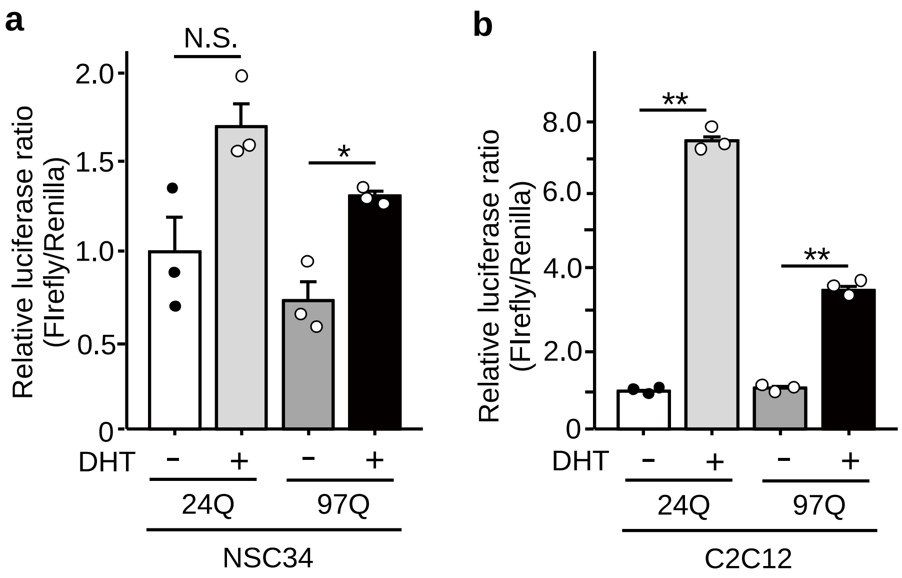

Supplementary Fig. 1. AR transcriptional activity reporter assay. (**a, b**) AR transcriptional activity in NSC34 (**a**) and C2C12 cells (**b**) stably expressing AR-24Q or AR-97Q treated with or without DHT. Quantitative analyses were performed on n=3 samples per group. Error bars indicate the s.e.m. *p<0.05 and **p<0.01, ANOVA with Tukey' test (**a, b**). DHT, dihydrotestosterone, N.S., not significant. Source data are provided as a Source Data file (**a, b**).

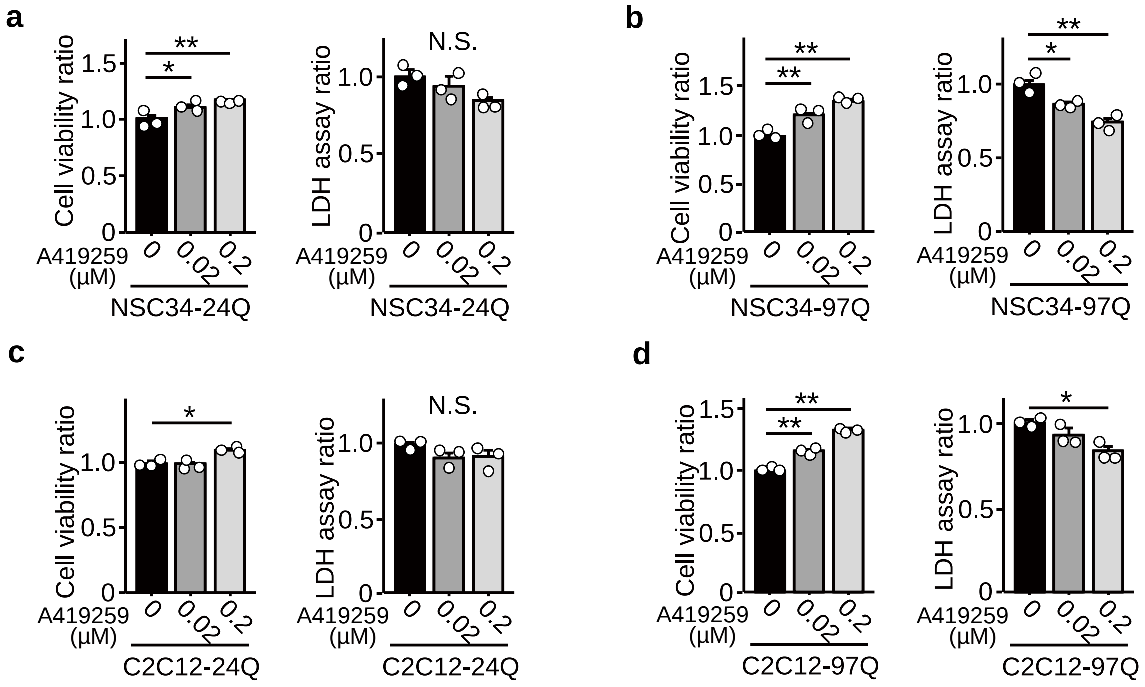

Supplementary Fig. 2. A419259 improves phenotypes of cellular models of SBMA in a dose dependent manner. (**a**-**d**) The viability and LDH release from NSC34 (**a**, **b**) and C2C12 cells (**c**, **d**) stably expressing AR-24Q or AR-97Q that were treated with or without A419259 are shown (n=3). Error bars indicate the s.e.m. *p<0.05 and **p<0.01, ANOVA with Dunnett's test (**a**-**d**). N.S., not significant. Source data are provided as a Source Data file (**a**-**d**).

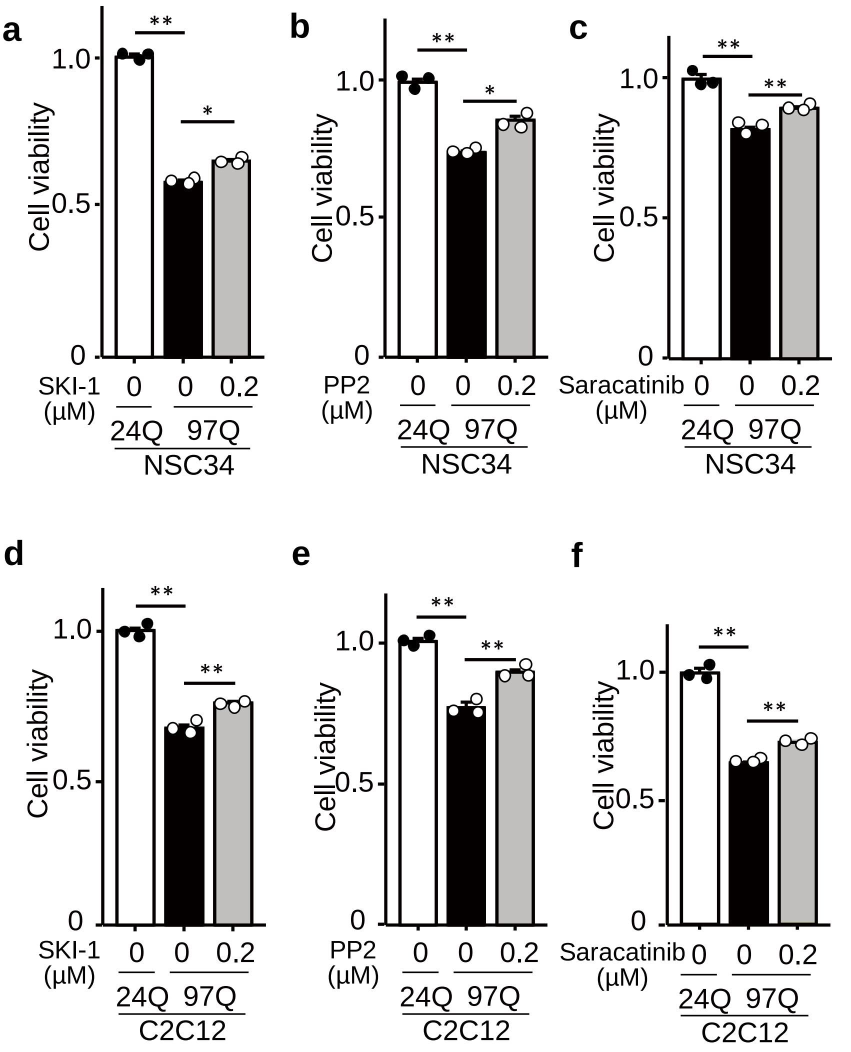

Supplementary Fig. 3. The effect of SKI on cellular models of SBMA. (**a**-**f**) The viability of NSC34 (**a**-**c**) and C2C12 cells (**d**-**f**) stably expressing AR-24Q or AR-97Q treated with or without SKI-1, PP2 or Saracatinib (n=3). Error bars indicate the s.e.m. *$p<0.05$ and **$p<0.01$, ANOVA with Dunnett's test (**a**-**f**). Source data are provided as a Source Data file (**a**-**f**).

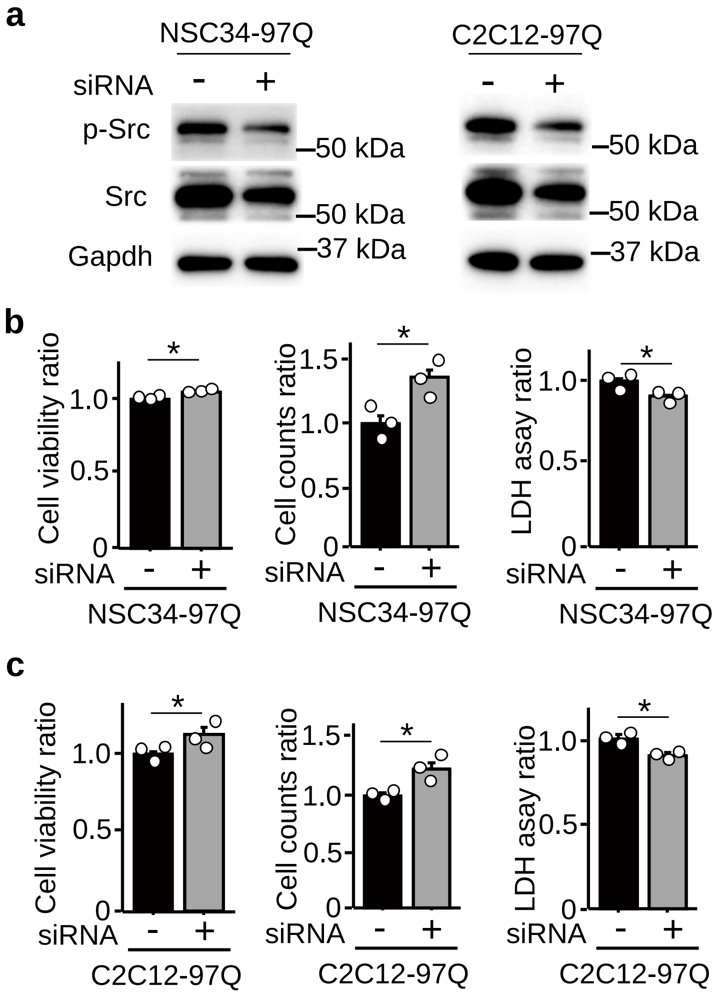

Supplementary Fig. 4. Suppression of Src phosphorylation improves viability of cellular models of SBMA. (**a**) Immunoblots showing the levels of the p-Src and Src proteins in DHT-treated NSC34 and C2C12 cells stably expressing AR-97Q and transfected with the mock or Src siRNA. (**b, c**) The viability, cell numbers and LDH release from NSC34 (**b**) and C2C12 cells (**c**) are shown (n=3). Error bars indicate the s.e.m. Statistical analyses were performed using the unpaired two-sided *t*-test (**b, c**). *p<0.05. Source data are provided as a Source Data file (**a-c**).

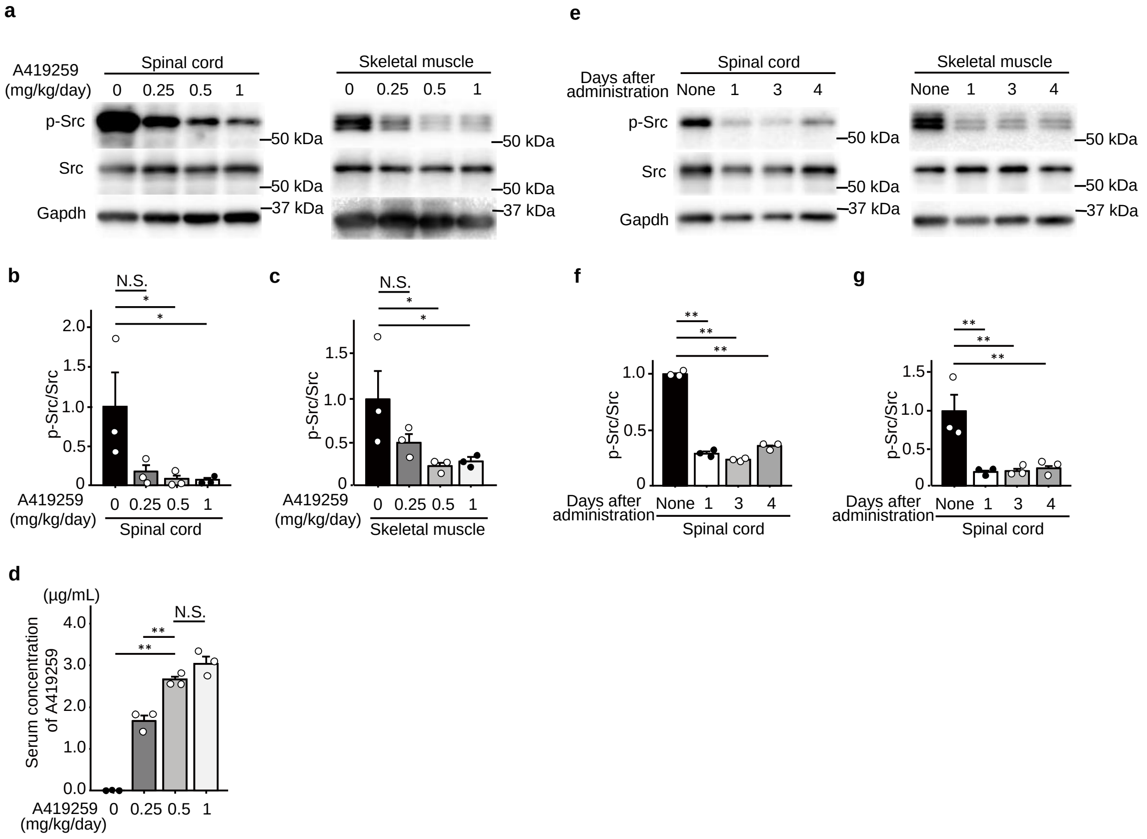

Supplementary Fig. 5. The administration of 0.5mg/kg/day A419259 to the mouse model of SBMA sufficiently suppresses Src phosphorylation. (**a**) Immunoblots for p-Src and Src in the spinal cord and skeletal muscle of 7-week-old AR-97Q mice treated with 0.25, 0.5 or 1mg/kg/day A419259 or vehicle. (**b, c**) Quantitative analyses were performed using densitometry (n=3 animals per group). (**d**) The concentration of A419259 in the serum of AR-97Q mice treated with 0.25, 0.5 or 1 mg/kg/day A419259 or without A419259 (n=3 animals per group). (**e**) Immunoblots for p-Src and Src in the spinal cord and skeletal muscle of 7-week-old AR-97Q mice one, three or four days after treated with 0.5mg/kg/day A419259. (**f, g**) Quantitative analyses were performed using densitometry (n=3 animals per group). Error bars indicate the s.e.m. *p<0.05 and **p<0.01, ANOVA with Dunnett's test (**b, c, d, f, g**). N.S., not significant. Source data are provided as a Source Data file (**a-g**).

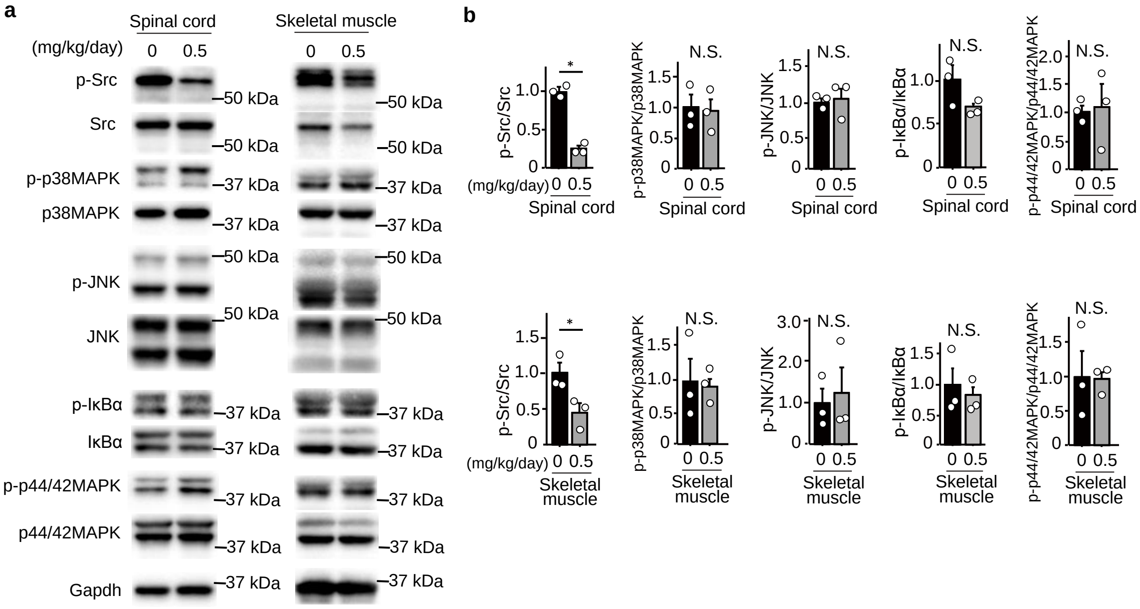

Supplementary Fig. 6. A419259 did not change the phosphorylation of p38MAPK, JNK, IκBα or p44/42MAPK in the mouse model of SBMA. (**a**) Immunoblots for phosphorylated and total p38MAPK, JNK, IκBα and p44/42MAPK in the spinal cord and skeletal muscle of 7-week-old AR-97Q mice treated with or without A419259. (**b**) Quantitative analyses were performed using densitometry (n=3 animals per group). Error bars indicate the s.e.m. *p<0.05, Statistical analyses were performed using the unpaired two-sided t-test (**b**). N.S., not significant. Source data are provided as a Source Data file (**a, b**).

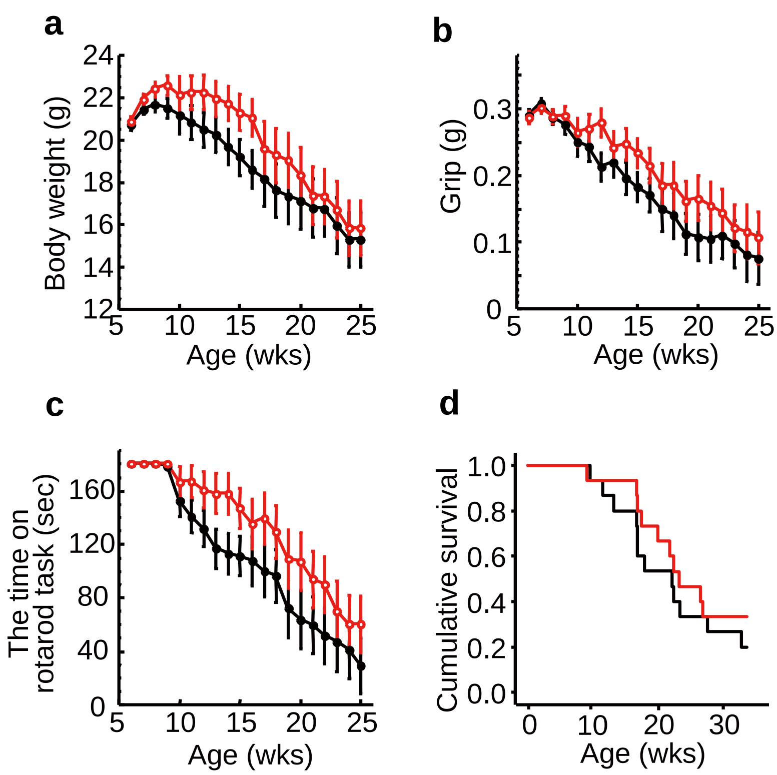

Supplementary Fig. 7. Effects of A419259 on the phenotypes of AR-97Q mice at 0.25mg/kg/day once in 3 days. (**a**-**d**) Body weight (**a**), grip power (**b**), rotarod task performance (**c**) and survival rate (**d**) of AR-97Q mice treated with or without A419259 (n = 15 per group). Black lines indicate vehicle-treated mice and red lines indicate A419259-treated mice. Not significant at 13 weeks, as determined by the unpaired two-sided *t*-test for body weight, grip power and rotarod performance; not significant by the log-rank test. Error bars indicate s.e.m. Source data are provided as a Source Data file (**a**-**d**).

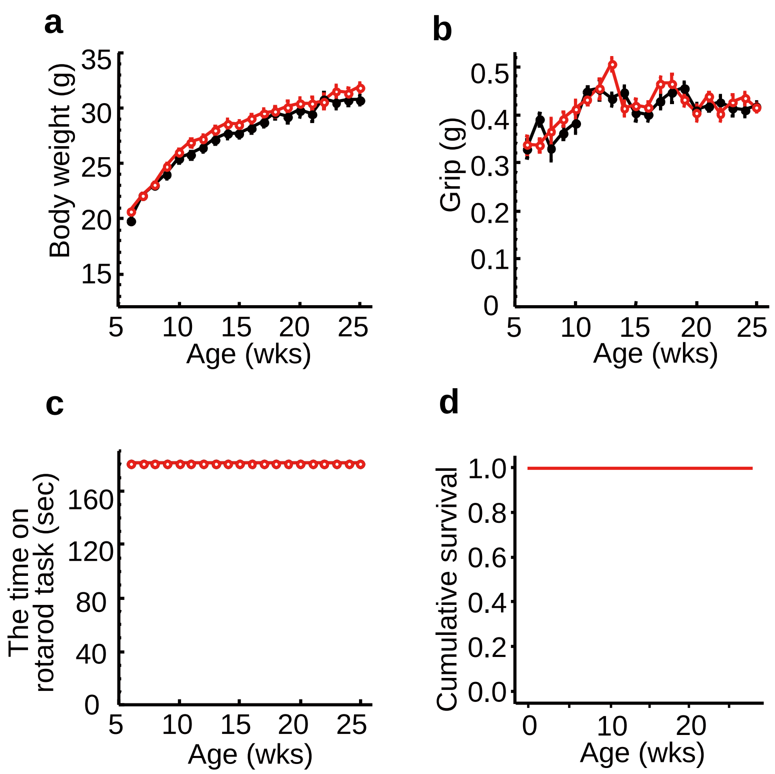

Supplementary Fig. 8. Effects of A419259 on the phenotypes of wild-type mice. (**a-d**) Body weight (**a**), grip power (**b**), rotarod task performance (**c**) and survival rate (**d**) of wild-type mice treated with or without A419259 at 0.5mg/kg/day once in 3 days (n=10 per group). Black lines indicate vehicle-treated mice and red lines indicate A419259-treated mice. Not significant at 13 weeks, as determined by the unpaired two-sided *t*-test for body weight, grip power and rotarod performance; not significant by the log-rank test. Error bars indicate s.e.m. Source data are provided as a Source Data file (**a-d**).

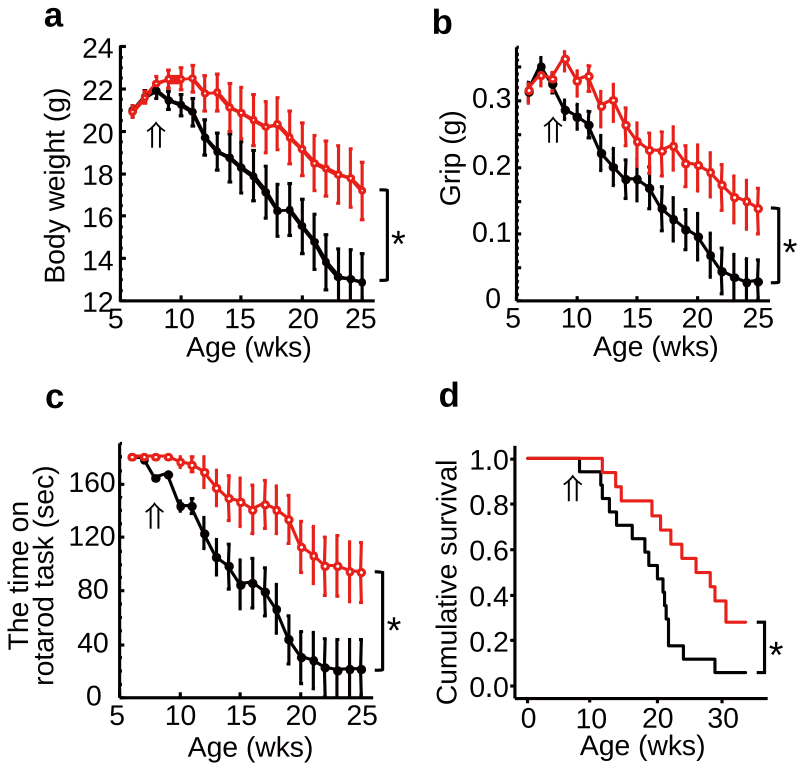

Supplementary Fig. 9. Effects of post-onset administration of A419259 on the phenotypes of AR-97Q mice. (**a**-**d**) Body weight (**a**), grip power (**b**), rotarod task performance (**c**) and survival rate (**d**) of AR-97Q mice treated with or without A419259 (n=17 for vehicle and n=16 for A419259). A419259 was administered from 8 weeks of age (arrows) until the end of the analysis. Error bars indicate s.e.m. *p<0.05 at 13 weeks by unpaired two-sided t-test for body weight, grip power and rotarod performance (**a**-**c**); and *p<0.05 by log-rank test (**d**). Black lines indicate vehicle-treated mice and red lines indicate A419259-treated mice (**a**-**d**). Source data are provided as a Source Data file (**a**-**d**).

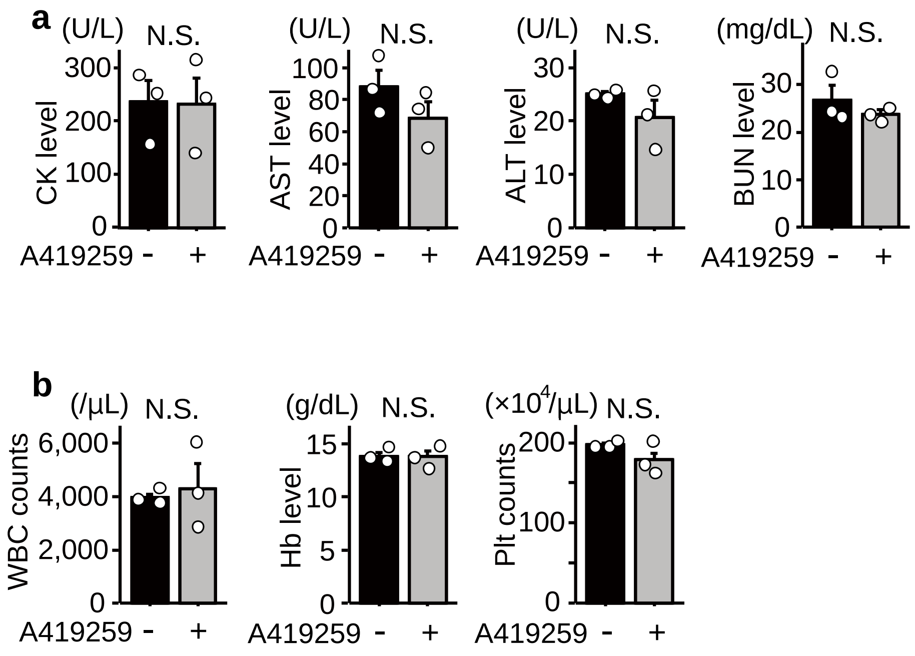

Supplementary Fig. 10. Hematological analyses of A419259-treated AR-97Q mice. (**a**) The creatine kinase (CK), aspartate aminotransferase (AST), alanine aminotransferase (ALT) and blood urea nitrogen (BUN) levels in the serum of AR-97Q mice treated with or without A419259 at 13 weeks of age (n=3). (**b**) The white blood cell (WBC), hemoglobin (Hb) and platelet (Plt) levels in the whole blood of AR-97Q mice treated with or without A419259 at 13 weeks of age (n=3). Error bars indicate s.e.m. An unpaired two-sided *t*-test (**a, b**) was used for comparison. N.S., not significant. Source data are provided as a Source Data file (**a, b**).

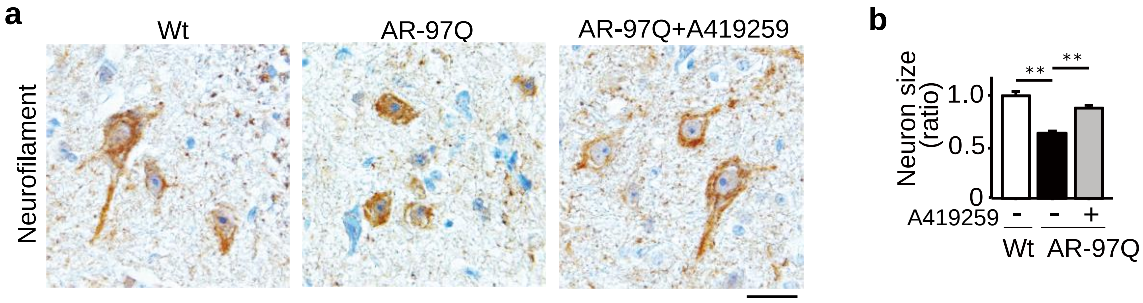

Supplementary Fig. 11. A419259 improves motor neuron size of SBMA mice. (**a**, **b**) Immunostaining for neurofilament heavy chain in spinal cord samples from 13-week-old mice (**a**) and quantitation of the motor neuron size (**b**). Quantitative analysis were performed on sections from n=3 animals per group. Error bars indicate the s.e.m. $^{**}p<0.01$. ANOVA with Dunnett's test was used for comparison (**b**). Scale bars: 25μm (**a**). Source data are provided as a Source Data file (**b**).

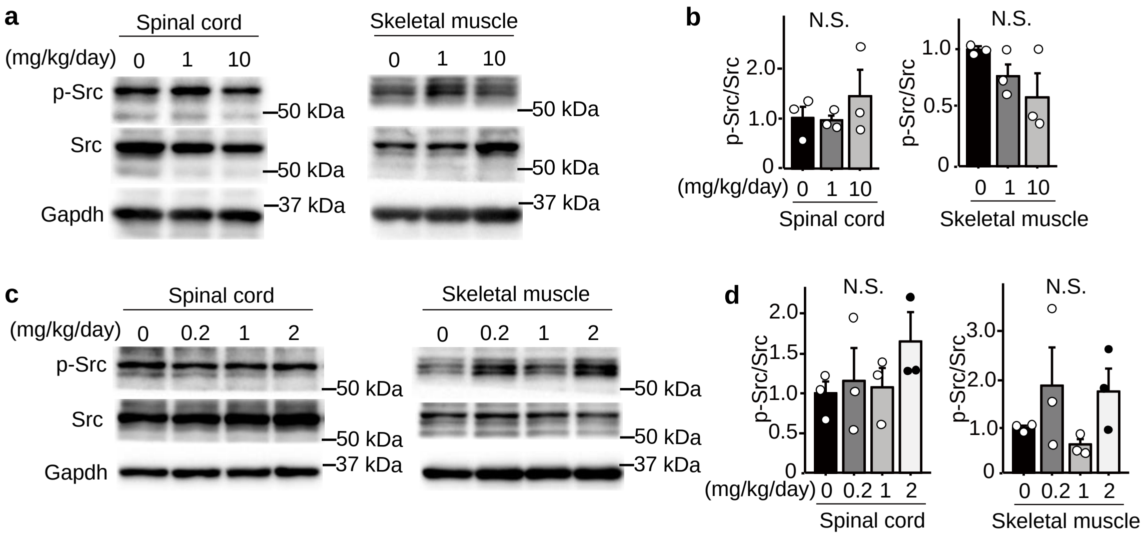

Supplementary Fig. 12. Effect of SKI-1 and PP2 on Src phosphorylation in the mouse model of SBMA. (**a**) Immunoblots for p-Src and Src in the spinal cord and skeletal muscle of 6-week-old AR-97Q mice treated with or without SKI-1. (**b**) Quantitative analyses were performed using densitometry (n=3 animals per group). (**c**) Immunoblots for p-Src and Src in the spinal cord and skeletal muscle of 6-week-old AR-97Q mice treated with or without PP2. (**d**) Quantitative analyses were performed using densitometry (n=3 animals per group). Error bars indicate the s.e.m. ANOVA with Dunnett's test was used for comparison (**b, d**). N.S., not significant. Source data are provided as a Source Data file (**a-d**).

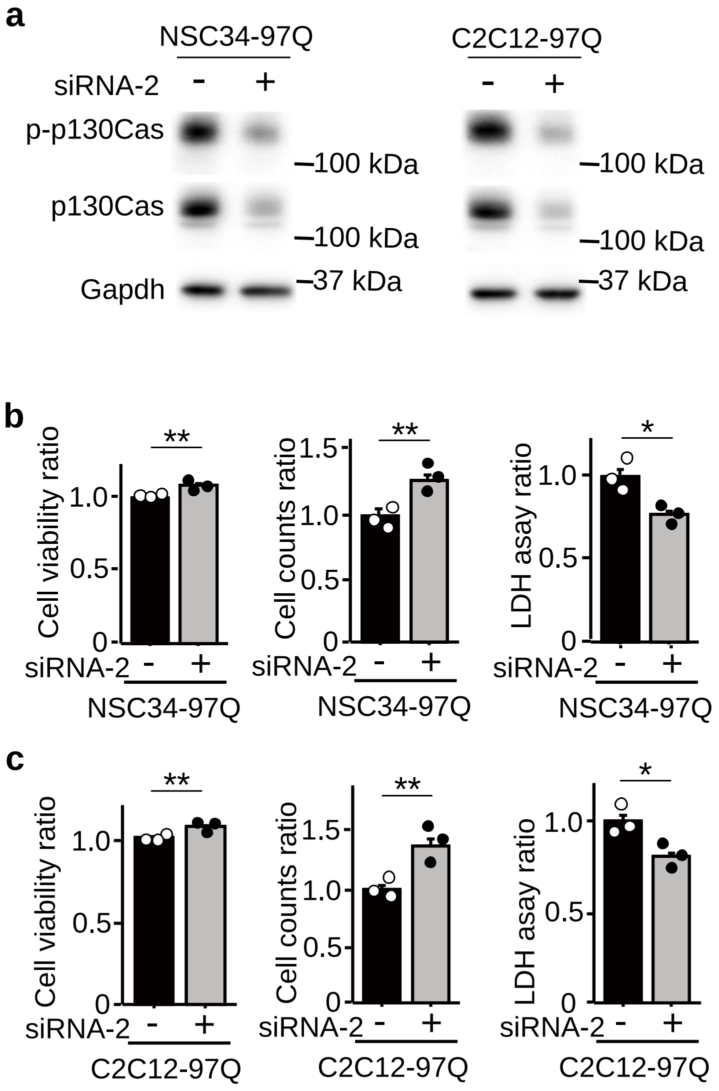

Supplementary Fig. 13. Down-regulation of phosphorylated p130Cas alleviated toxic effects on cellular models of SBMA. (**a**) Immunoblots showing the levels of the p-p130Cas and p130Cas proteins in DHT-treated NSC34 and C2C12 cells stably expressing AR-97Q and transfected with the mock or p130Cas siRNA-2. (**b, c**) The viability, cell numbers and LDH release from NSC34 (**b**) and C2C12 cells (**c**) are shown (n=3). Error bars indicate the s.e.m. Statistical analyses were performed using the unpaired two-sided t-test (**b, c**). *$p<0.05$ and **$p<0.01$. Source data are provided as a Source Data file (**a-c**).

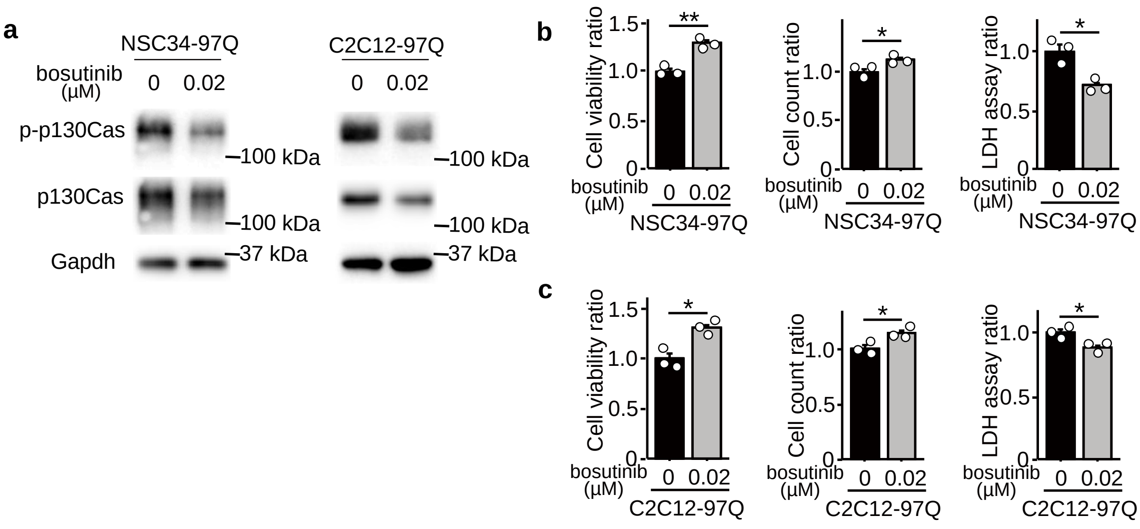

Supplementary Fig. 14. Effect of bosutinib in cellular models of SBMA. (**a**) Immunoblots showing the levels of p-p130Cas and p130Cas proteins in DHT-treated NSC34 and C2C12 cells stably expressing AR-97Q and treated with or without bosutinib. (**b, c**) The viability, cell counts and LDH release of NSC34 (**b**) and C2C12 cells (**c**) stably expressing AR-97Q treated with or without bosutinib (n=3). Error bars indicate the s.e.m. *$p<0.05$ and **$p<0.01$. An unpaired two-sided t-test (**b, c**) was used for comparison. Source data are provided as a Source Data file (**a-c**).

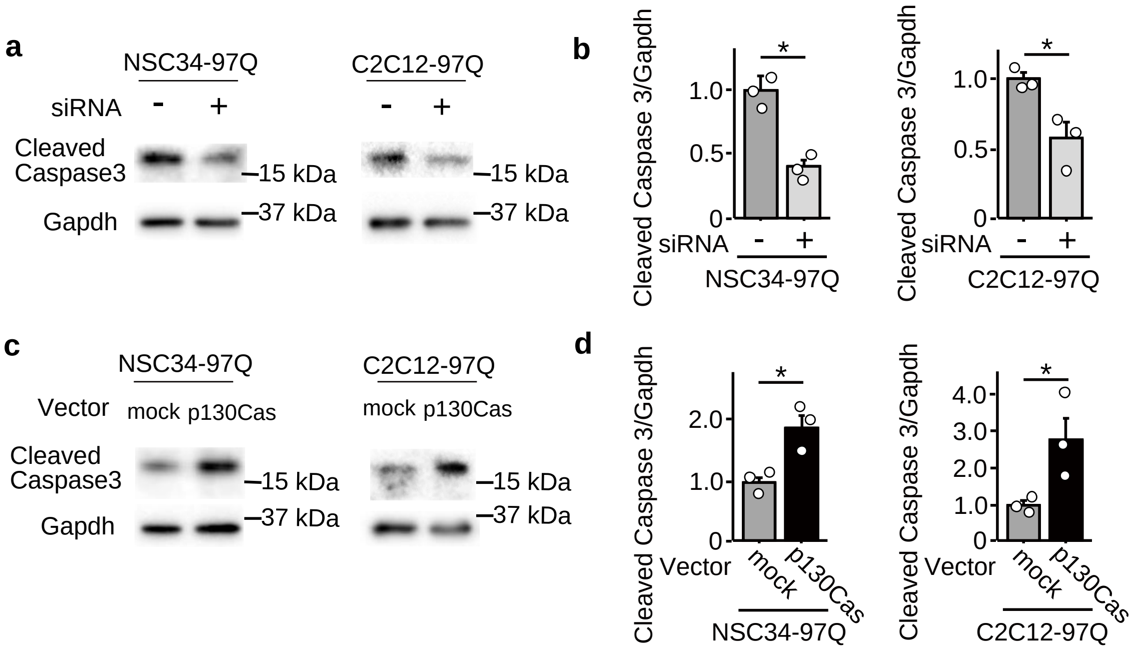

Supplementary Fig. 15. Overexpression of p130Cas induces caspase 3 activation. (**a**) Immunoblots for cleaved caspase3 protein in DHT-treated NSC34 and C2C12 cells stably expressing AR-97Q and transfected with the mock or p130Cas siRNA. (**b**) Quantitative analyses were performed using densitometry (n=3). (**c**) Immunoblots for cleaved caspase 3 protein in DHT-treated NSC34 and C2C12 cells stably expressing AR-97Q and transfected with the mock or p130Cas vector. (**d**) Quantitative analyses were performed using densitometry (n=3). Error bars indicate the s.e.m. *$p<0.05$. An unpaired two-sided *t*-test was used for comparison (**b, d**). Source data are provided as a Source Data file (**a-d**).

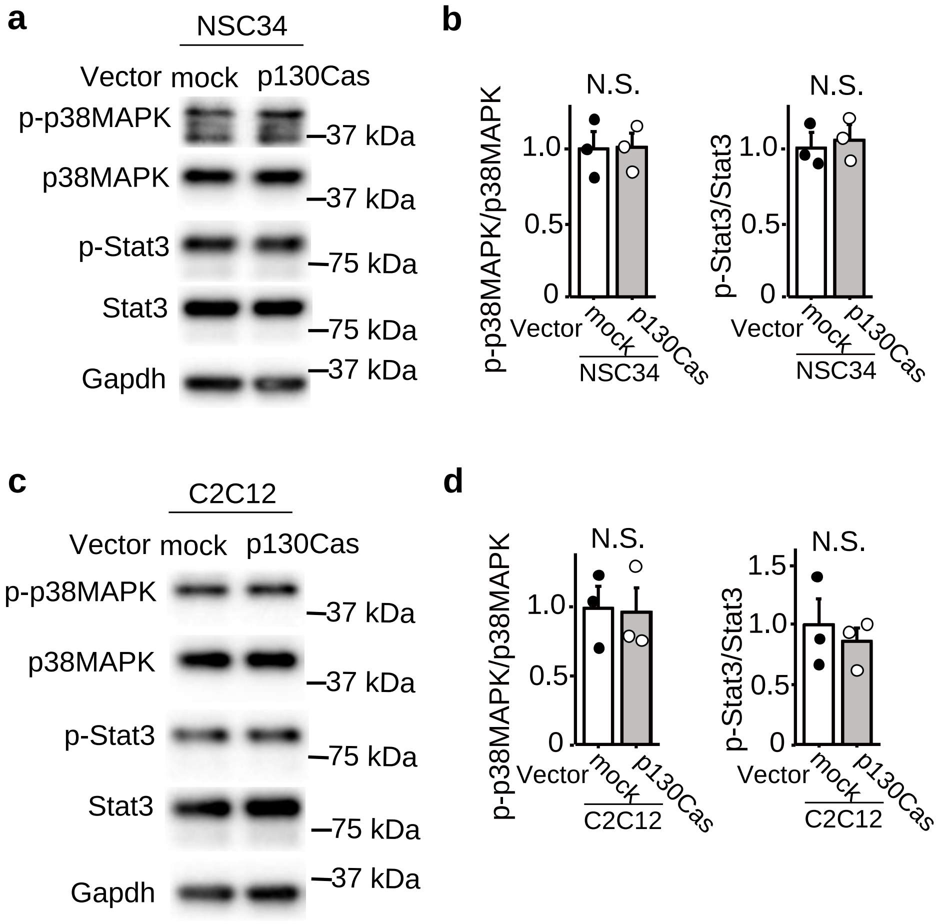

Supplementary Fig. 16. Relationship between p130Cas and p38MAPK or Stat3. (a-d) Immunoblots for phosphorylated and total p38MAPK and Stat3 in the NSC34 (**a**) and C2C12 (**c**) cells transfected with mock or p130Cas vector. Quantitative analysis was performed using densitometry (n=3) (**b, d**). Error bars indicate s.e.m. An unpaired two-sided *t*-test (**b, d**) was used for comparison. N.S., not significant. Source data are provided as a Source Data file (**a-d**).

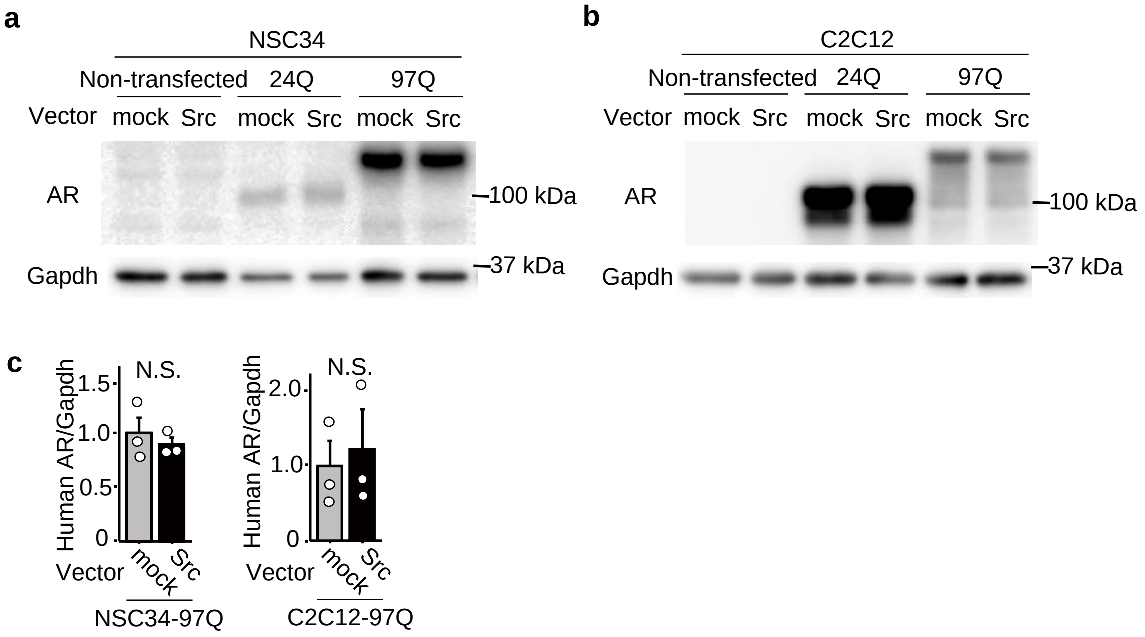

Supplementary Fig. 17. Effect of Src on AR protein levels in the cellular models of SBMA. (**a**-**c**) Immunoblots for human AR in the NSC34 (**a**) and C2C12 (**b**) stably expressing AR-24Q or AR-97Q with DHT transfected mock or Src vector. Quantitative analysis was performed using densitometry (n=3) (**c**). Error bars indicate s.e.m. An unpaired two-sided *t*-test (**c**) was used for comparison. N.S., not significant. Source data are provided as a Source Data file (**a**-**c**).

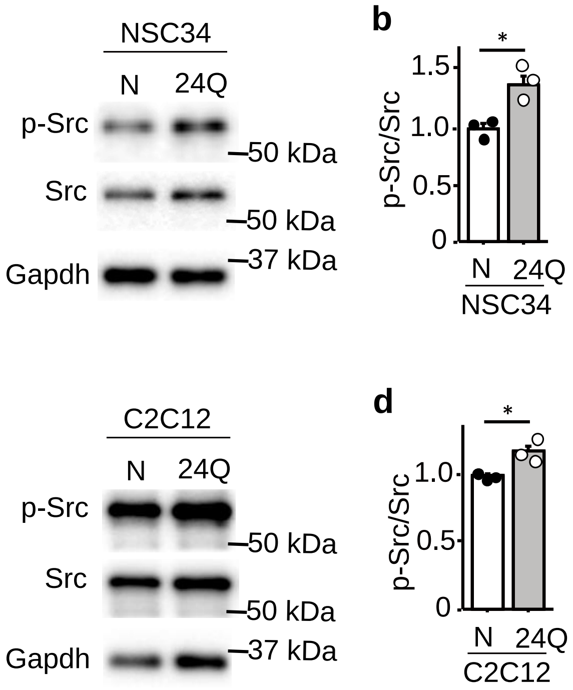

Supplementary Fig. 18. Src phosphorylation in neuronal and muscular cells expressing AR-24Q. (**a**-**d**) Immunoblots for phosphorylated and total p38MAPK and Stat3 in the NSC34 (**a**) and C2C12 (**c**) cells stably expressing AR-24Q transfected with mock or p130Cas vector. Quantitative analysis was performed using densitometry (n=3) (**b**, **d**). Error bars indicate s.e.m. Statistical analyses were performed using the unpaired two-sided *t*-test (**b**, **d**). *p<0.05 and **p<0.01. N, non-transfected. Source data are provided as a Source Data file (**a**-**d**).

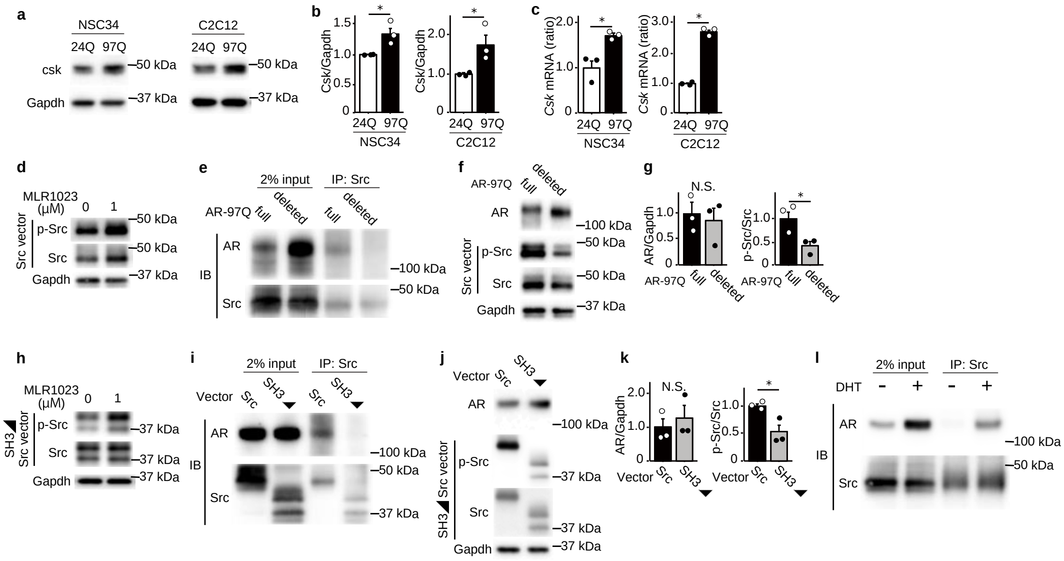

Supplementary Fig. 19. Src is activated by binding with AR-97Q. (**a**) Immunoblots showing the levels of Csk protein in NSC34 or C2C12 cells stably expressing AR-24Q or AR-97Q. (**b**) Quantitative densitometry analysis of Csk protein levels in NSC34 or C2C12 cells stably expressing AR-24Q or AR-97Q. (**c**) The mRNA levels of *Csk* in NSC34 or C2C12 cells stably expressing AR-24Q or AR-97Q. (**d**) Immunoblots showing the levels of the p-Src protein in NSC34 cells that were transfected with a Src vector and treated with or without a Src activator MLR1023. (**e**) NSC34 cells transfected with the Src vector and the full length of AR-97Q or deleted AR-97Q vector were immunoprecipitated with an anti-Src antibody and immunoblotted with an antibody against AR. (**f**) Immunoblots showing the levels of the AR, p-Src and Src proteins in NSC34 cell co-transfected with the Src vector and the full length of AR-97Q or deleted AR-97Q vector. (**g**) Quantitative densitometry analysis of AR and p-Src levels in NSC34 cells co-transfected with the Src vector and the full length of AR-97Q or deleted AR-97Q vector. (**h**) Immunoblots showing the levels of the p-Src protein in NSC34 cells that were transfected with SH3 deleted-Src (SH3◢ Src) vector and treated with or without the Src activator. (**i**) NSC34 cells stably expressing AR-97Q and transfected with the Src or SH3◢ Src vector were immunoprecipitated with a Src antibody and immunoblotted with an antibody against AR. (**j**) Immunoblots showing the levels of the AR, p-Src and Src proteins in the AR-97Q cells that were transfected with the Src or SH3◢ Src vector. (**k**) Quantitative densitometry analysis of AR and p-Src levels in the AR-97Q cells transfected with the full-length Src or SH3◢ Src vector. (**l**) NSC34 cells stably expressing AR-97Q transfected with the Src vector and treated with ethanol or DHT were immunoprecipitated with a Src antibody and immunoblotted with an AR antibody. Error bars indicate s.e.m. n=3. Statistical analyses were performed using the unpaired two-sided t-test (**b, c, g, k**). *p<0.05. N.S., not significant. Source data are provided as a Source Data file (**a-l**).

Supplementary Table 1. List of used primers.

Genotyping primers

Mice	Forward (5'-3')	Reverse (5'-3')
AR-97Q	CTTCTGGCGTGTGACCGGCG	TGAGCTTGGCTGAATCTTCC

Site-directed mutagenesis primers

Vector	Forward (5'-3')	Reverse (5'-3')
Deleted AR-97Q	AAGCTGGAGAACCCGCTGGAC TACG	TCCGGCCAGAGCCAGTGGAAAG TTG

RT-PCR primers

Gene	Forward (5'-3')	Reverse (5'-3')
Csk	GAGGAGAAGGGTGGGCTCTA	TTCCCGAAGCTGCAAAAACG
Src	TTAGGCCAAGACCCCGGATA	AGTTGCTGGGGATGTAACCG
Gapdh	CGTCCCGTAGACAAAATGGT	GAATTTGCCGTGAGTGGAGT
